# Long non-coding RNA LINC00704 promotes cell proliferation, migration, and invasion in papillary thyroid carcinoma via miR-204-5p/HMGB1 axis

**DOI:** 10.1515/biol-2020-0057

**Published:** 2020-08-12

**Authors:** Yihui Lin, Jianjia Jiang

**Affiliations:** Department of Endocrinology, The Quanzhou First Hospital Affiliated to Fujian Medical University, Chongfu Building D-202, Dongjie, Quanzhou City 362000, Fujian, China

**Keywords:** LINC00704, miR-204-5p, HMGB1, papillary thyroid carcinoma

## Abstract

Papillary thyroid carcinoma (PTC) is a common malignancy worldwide. LncRNA LINC00704 (mitotically associated long non-coding RNA) was reported as a crucial regulator in PTC. However, the biological mechanism of LINC00704 action remains unclear in PTC. The mRNA levels of LINC00704, miR-204-5p, and high-mobility group box 1 (HMGB1) were measured by quantitative reverse transcription-polymerase chain reaction (qRT-PCR) assay. HMGB1, proliferating cell nuclear antigen (PCNA), and cyclin D1 protein levels were detected using the Western blot assay. The binding relationship between miR-204-5p and LINC00704 or HMGB1 was predicted by LncBase Predicted v.2 or TargetScan, respectively, and then validated by dual luciferase reporter assay. Cell viability, cell cycle, cell migration and invasion, and migration ratio were assessed by MTT, flow cytometry, transwell cell migration and invasion, and wound-healing assays, respectively. Results suggested that LINC00704 and HMGB1 were elevated and miR-204-5p decreased in PTC tissues and cells. Furthermore, rescue experiments demonstrated that the miR-204-5p inhibitor alleviated the inhibitory effects of LINC00704 knockdown on cell proliferation, cell cycle, migration, and invasion. Meanwhile, miR-204-5p overexpression repressed proliferation, migration, and invasion by targeting HMGB1. Mechanical analysis discovered that LINC00704 could act as an miR-204-5p sponge to modulate HMGB1 expression. In conclusion, LINC00704 promoted PTC cell proliferation, cell cycle, migration, and invasion by the miR-204-5p/HMGB1 axis, providing a novel therapeutic target for PTC patients.

## 1 Introduction

Papillary thyroid carcinoma (PTC) is a common endocrine system tumor and accounts for 80–90% of thyroid cancer [1]. The morbidity of PTC has been increasing over the last two decades [2]. PTC easily metastasizes to the cervical lymph nodes, and this may be fatal for PTC patients [3]. Therefore, it is crucial to develop a new therapeutic target for PTC patients in the early stage of diagnosis.

Long non-coding RNAs (lncRNAs) are a class of non-coding RNAs with more than 200 nucleotides (nts) in length and can regulate gene expression at the transcriptional and posttranscriptional levels [4]. LncRNA dysregulation has been reported in many cancer tissues, and their abnormal expression may relate to cancer progression. For instance, lncRNA nuclear-enriched abundant transcript 1 (NEAT1) was dramatically increased in PTC tissues and cell lines, and its overexpression promoted cell proliferation, invasion, migration, and induced cell apoptosis; and the depletion of NEAT1 restrained xenograft tumor growth [5]. Previous studies documented that the level of LINC00704 was upregulated in PTC; LINC00704 knockdown restrained cell proliferation, migration, invasion, cell colony formation ability, and induced cell apoptosis in PTC [6]. However, the biological mechanism of LINC00704 action remains undefined in PTC.

MicroRNAs (miRNAs), a class of non-coding RNAs of about 22 nts in length, have been reported to function as messenger RNA (mRNA) inhibitors by downregulating mRNA translation or mediating mRNA degradation [7]. Also, the dysregulation of miRNA has been reported in many cancer processes such as cancer initiation, progression, and transition. For example, miR-23a was markedly reduced in PTC tissues and cells; the overexpression of miR-23a significantly impeded cell proliferation, induced cell cycle arrest at G0/G1 phase, and promoted cell apoptosis, while an miR-23a inhibitor showed the opposite effects [8]. Another study in osteosarcoma demonstrated that miR-204-5p was conspicuously downregulated in osteosarcoma tissues and osteosarcoma cell lines; miR-204-5p overexpression promoted cell apoptosis and inhibited cell migration and invasion; and miR-204-5p mimics hampered the xenograft tumor growth *in vivo* [9]. High-mobility group box 1 (HMGB1) is a ubiquitously expressed intracellular protein that binds DNA and transcription factors and regulates chromosomal structure and function [10]. HMGB1 has been identified as a crucial oncogene in several cancer types. HMGB1 was highly expressed in many cancer tissues and/or cells including prostate cancer [11], bladder cancer [12], human non-small cell lung cancer [13], gastric cancer [14], colon cancer [15], and also in PTC [16,17]. However, the biological mechanisms of miR-204-5p and HMGB1 action were still unclear in PTC.

In this study, we verified that LINC00704 and HMGB1 were distinctly upregulated, and miR-204-5p was drastically downregulated in PTC tissues and cells. Furthermore, we found that LINC00704 modulated HMGB1 to regulate cell proliferation, migration, and invasion in PTC by sponging miR-204-5p. This new regulatory pathway may provide a novel molecular target for early stage PTC diagnosis.

## 2 Materials and methods

### 2.1 Tissue samples

Fifty PTC tissues and the corresponding adjacent normal tissues were collected from the Quanzhou First Hospital Affiliated to Fujian Medical University. All tissues were frozen at −80°C until further use.


**Informed consent:** Informed consent has been obtained from all individuals included in this study.
**Ethical approval:** The research related to human use has been complied with all the relevant national regulations, institutional policies and in accordance with the tenets of the Helsinki Declaration, and has been approved by the Ethics Committee of the Quanzhou First Hospital Affiliated to Fujian Medical University.

### 2.2 Cell culture and transfection

Four PTC cell lines (TPC-1, BCPAP, BHT101, and K1) and human thyroid epithelial cells (HTori-3) were purchased from Cell Bank of Chinese Academy of Sciences (Shanghai, China). All cells were cultured in RPMI-1640 medium (Invitrogen, Carlsbad, CA, USA) supplemented with 10% fetal bovine serum (FBS; Thermo Fisher Scientific, Rockville, MD, USA) and 1% penicillin/streptomycin (Invitrogen). The cells were cultivated in an incubator with the parameters of 37°C and 5% CO_2_. Small interfering RNA target for LINC00704 (si-LINC00704) and its matched control (si-NC); LINC00704 overexpression vector (LINC00704) and its matched control (vector); miR-204-5p mimic and miR-NC; miR-204-5p inhibitor and anti-miR-NC; and HMGB1 overexpression vector (HMGB1) and its matched control were obtained from Origene (Rockville, MD, USA). The transfection was conducted using Lipo-fectamine 2000 Reagent (Invitrogen) in accordance with the manual.

### 2.3 Quantitative reverse transcription- polymerase chain reaction (qRT-PCR)

The miRNeasy Mini Kit (Qiagen, Valencia, CA, USA) was used to extract RNA from cells, and the RNA samples were reverse transcribed using Transcriptor First Strand cDNA Synthesis Kit (Roche, Vilvoord, Brussel, Belgium). Quantitative PCR was conducted using FastStart Universal SYBR Green Master (Roche) by ABI Prism 7700 Sequence Detection System (Thermo Fisher Scientific). The data were calculated by using the 2^−ΔΔCt^ method, normalizing with endogenous control glyceraldehyde 3-phosphate dehydrogenase (GAPDH) and U6. All the primers were obtained from Beijing Genomics Institute (BGI, Shenzhen, China) and are listed as follows: LINC00704: forward 5′-TGCGTTCAGTAAAACGGGCA-3′, reverse 5′-TGTGGGAAATGCAGGGTTCT-3′; miR-204-5p: forward 5′-GACGCTTTCCCTTTGTCATCCT-3′, reverse 5′-GTGCAGGGTCCGAGGTATTC-3′; HMGB1: forward 5′-AGGATCCCAATGCACCCAAG-3′, reverse 5′-CGCAACATCACCAATGGACAG-3′; GAPDH: forward 5′-CGAGATCCCTCCAAAATCAA-3′, reverse 5′-TTCACACCCATGACGAACAT-3′; U6: forward 5′-CTCGCTTCGGCAGCACA-3′, reverse 5′-AACGCTTCACGAATTTGCGT-3′.

### 2.4 Western blot

Protein was extracted using a Protein Extraction Kit (Beyotime, Shanghai, China), and the sample concentration was detected using bicinchoninic acid (BCA) Protein Assay Kit (Beyotime). Following separation by sodium dodecyl sulfate–polyacrylamide gel electrophoresis (SDS-PAGE), the sample was transferred onto a polyvinylidene difluoride (PVDF) membrane (GE Healthcare, Piscataway, NJ, USA). Subsequently, the membrane was blocked in non-fat milk and incubated with primary antibody and secondary antibody in sequence. All antibodies were purchased from Abcam (Cambridge, MA, USA). The chemiluminescence intensity was assessed using eyoECL Plus Kit (Beyotime).

### 2.5 3-(4,5-dimethyl-2-thiazolyl)-2,5-diphenyl-2-*H*-tetrazolium bromide (MTT) assay

For MTT assay, 5 × 10^3^ cells were added into 96-well plates and cultivated for 24, 48, and 72 h. Then the cells were incubated with MTT for 3 h, and the formazan in the sample was dissolved by dimethyl sulfoxide (DMSO) for 15 min at 37°C in the dark. The absorbance was detected at 570 nm using a spectrophotometer (Thermo Fisher Scientific).

### 2.6 Cell cycle assay

In this assay, the transfected TPC-1 and BCPAP cells were trypsinized and washed with PBS, followed by fixation with 75% ethanol at 4°C overnight. Then the cells were centrifuged and resuspended in propidium iodide, followed by incubation for another 30 min. Thereafter, ModFit LT 3.0 for Windows (Verity Software House, Topsham, ME, USA) was applied to analyze the distribution of cells.

### 2.7 Transwell assay

For the migration assay, 500 µL of RPMI-1640 medium containing 10% FBS was added to the lower chamber, while the cells suspended in serum-free medium were added to the upper chamber. After incubation, the cells in the lower chamber were stained with 0.1% crystal violet. Cell numbers in 10 fields were counted using a light microscope and calculated using Image Pro Plus (Media Cybernetics, Silver Spring, MD, USA). The protocol of invasion assay was similar to that of the migration assay, while the difference being that the upper chamber was covered with a matrigel matrix (BD, Franklin Lakes, NJ, USA).

### 2.8 Wound-healing assay

In this assay, the PTC cells (1 × 10^4^ cells/well) were introduced into 96-well plates and then the starved monolayer cells were mounted on a reusable template to create a standard wound using a Wound Maker tool (Essen BioScience, Ann Arbor, MI, USA). Subsequently, an IncuCyte ZOOM Live-Cell Imaging System (Essen BioScience) was used to scan the plates at 0–24 h, and the IncuCyte ZOOM Software (Essen BioScience) was used to generate the quantified time-lapse curves in line with the operation manual.

### 2.9 Dual luciferase reporter assay

The interaction between LINC00704 and miR-204-5p was predicted by LncBase Predicted v.2 (http://carolina.imis.athena-innovation.gr), and the interaction between miR-204-5p and HMGB1 was predicted by TargetScan (http://www.targetscan.org). LINC00704 and HMGB1-3′UTR or their corresponding mutant sequences were amplified and inserted into a psiCHECK2 plasmid (Promega, Madison, WI, USA), namely, LINC00704-WT, LINC00704-MUT, HMGB1-WT and HMGB1-MUT. Following the transfection of miR-204-5p mimics or miR-NC in TPC-1 and BCPAP cells with a luciferase reporter, the luciferase activity was assessed using a Dual Luciferase Reporter Assay Kit (Promega). Renilla luciferase activities were used as the internal reference to normalize the firefly luciferase activities.

### 2.10 Statistical analysis

In this study, all quantitative data were repeated at least three times and reported as mean ± standard deviation. The differences between two groups were analyzed using Student's *t* test, while the differences among more than three groups were assessed by one-way analysis of variance (ANOVA). All data were calculated using GraphPad Prism 7 (GraphPad, La Jolla, CA, USA). A difference was considered statistically significant when *p* < 0.05.

## 3 Results

### 3.1 LINC00704 is upregulated in PTC tissues, cells, and correlated with the pathological characteristics of patients

**Figure 1:**
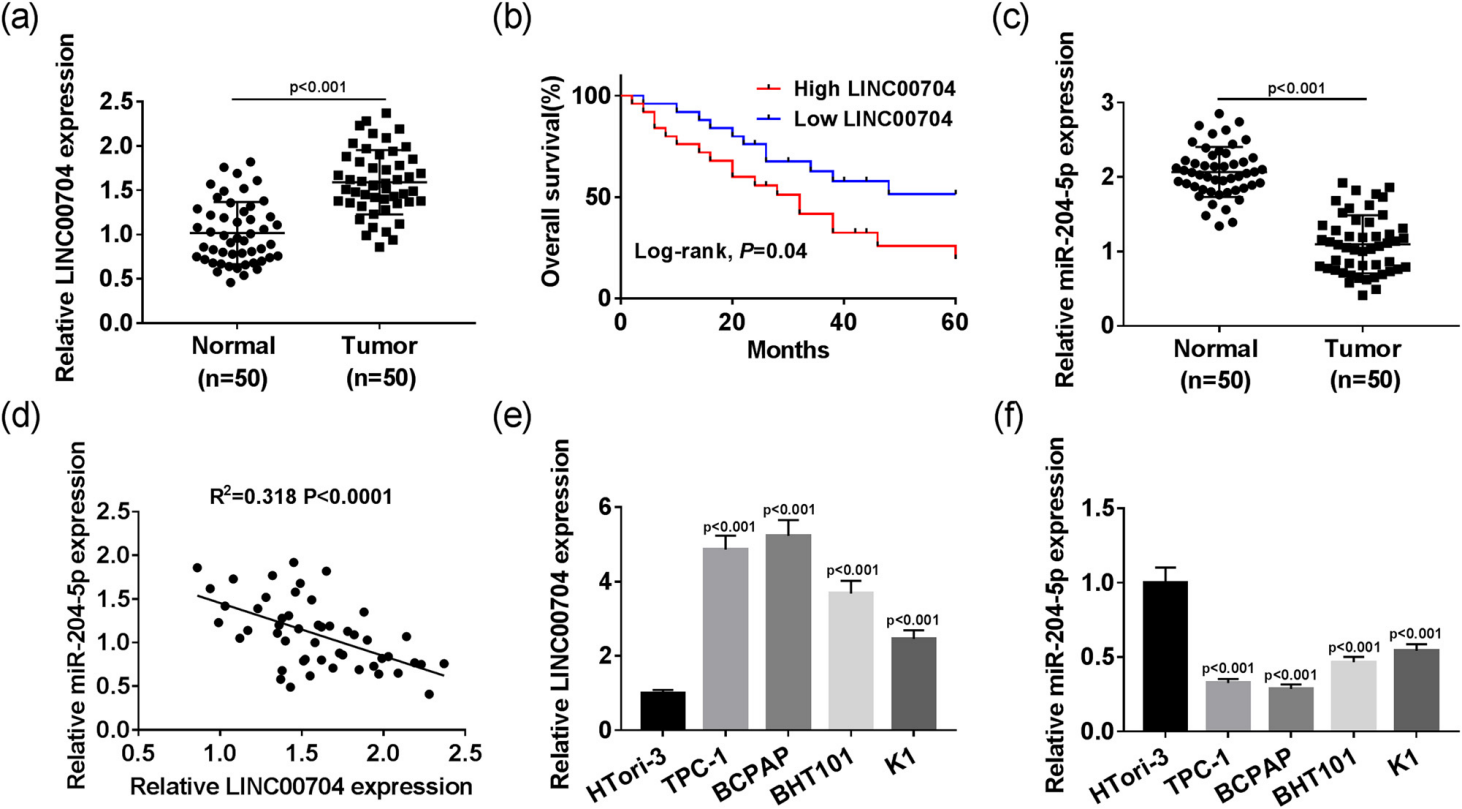
The relative expression of LINC00704 and miR-204-5p in PTC tissues and cells. The levels of LINC00704 (a and e) and miR-204-5p (c and f) were detected by qRT-PCR assay in PTC tissues and cells. (b) The overall survival ratio in high LINC00704 and low LINC00704 patients. (d) The correlation between miR-204-5p and LINC00704.

To study the effects of LINC00704 on PTC, qRT-PCR was first conducted to detect the expression of LINC00704 in PTC tissues and cells. The results showed that the relative expression of LINC00704 was significantly increased in PTC tissues and cells (TPC-1, BCPAP, BHT101, and K1) compared with that in the corresponding adjacent normal tissues or human thyroid epithelial cells (HTori-3), respectively (Figure 1a and e). Then the correlation analysis measured the relation between the level of LINC00704 and the clinical and pathological characteristics of PTC patients. The chi-square test results indicated that the high level of LINC00704 was closely correlated with tumor size (*P* = 0.015), TNM stage (*P* = 0.005), and lymph node metastasis (*P* = 0.024) but not correlated with age or gender (Table 1). In addition, patients with higher expression of LINC00704 had lower survival rates and *vice versa* (Figure 1b). Taken together, the level of LINC00704 was dramatically elevated in PTC tissues and cells and correlated with the pathological characteristics of patients including tumor size, TNM stage, and lymph node metastasis.

**Table 1:** LINC00704 expression and clinicopathologic characteristics in PTC

Parameters	Total	LINC00704 expression		*P* value
		High (*n* = 25)	Low (*n* = 25)	
Age				
<60	23	11	12	0.714
≥60	27	14	13	
Gender				
Male	17	10	7	0.413
Female	33	15	18	
Tumor size (cm)				
<1	23	8	15	0.015*
≥1	27	17	10	
TNM stage				
I–II	37	15	22	0.005*
III–IV	13	10	3	
Lymph node metastasis				
No	29	12	17	0.024*
Yes	21	13	8	

### 3.2 miR-204-5p is downregulated in PTC tissues, cells, and negatively linearly correlated with LINC00704

To study the roles of miR-204-5p in PTC, qRT-PCR was conducted to detect the expression of miR-204-5p in PTC tissues and cells. The results showed that miR-204-5p was remarkably downregulated in PTC tissues and cells in comparison with that in normal tissues and cells (Figure 1c and f). Additionally, the level of miR-204-5p was negatively linearly correlated with the level of LINC00704 (Figure 1d). These data revealed that miR-204-5p levels were reduced in PTC tissues and cells and negatively linearly correlated with LINC00704.

### 3.3 miR-204-5p is a target of LINC00704

**Figure 2:**
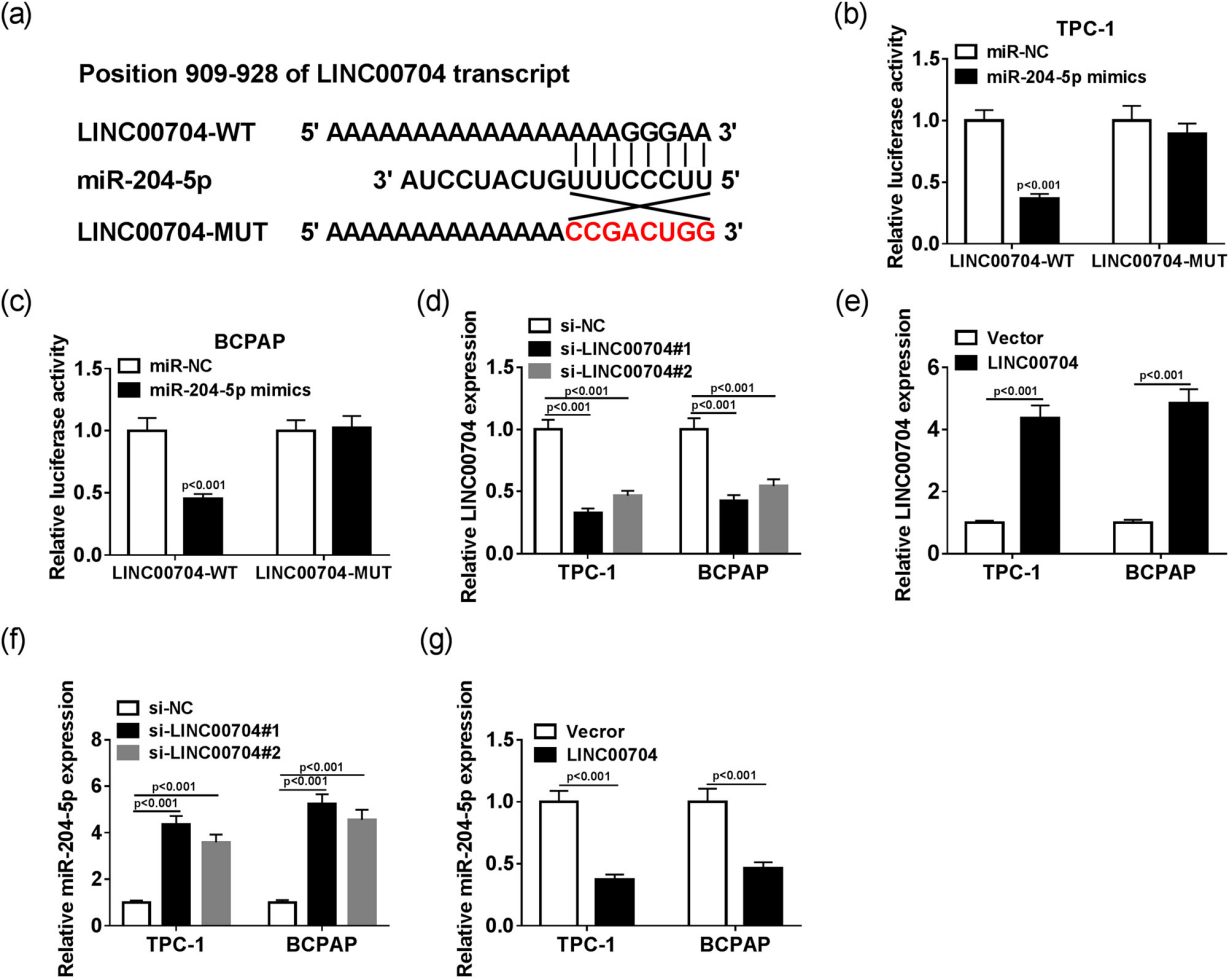
miR-204-5p is a target of LINC00704. (a) The putative complementary sequences between miR-204-5p and LINC00704. The luciferase activity of LINC00704-WT or LINC00704-MUT reporter in TPC-1 (b) and BCPAP (c) cells transfected with miR-204-5p mimics or miR-NC was assessed by dual luciferase reporter assay. The level of LINC00704 was detected by qRT-PCR in TPC-1 and BCPAP cells transfected with si-LINC00704 (d) or pcDNA-LINC00704 (e). The level of miR-204-5p was measured by qRT-PCR in TPC-1 and BCPAP cells transfected with si-LINC00704 (f) or pcDNA-LINC00704 (g).

To explore the biological role of LINC00704, LncBase Predicted v.2 online website was used to predict the targets of LINC00704. The results displayed that miR-204-5p shared complementary sequences with LINC00704 (Figure 2a). The dual luciferase reporter assay indicated that the transfection of miR-204-5p mimics lead to the apparent downregulation of the luciferase activity of LINC00704-WT but had no significant effect on the luciferase activity of LINC00704-MUT in TPC-1 and BCPAP cells (Figure 2b and c). Subsequently, the loss and gain assay indicated that the level of LINC00704 was conspicuously decreased and the level of miR-204-5p was notably elevated in TPC-1 and BCPAP cells transfected with si-LINC00704 and *vice versa* in the LINC00704 overexpressed group (Figure 2d–g). These results reveal that miR-204 negatively interacts with LINC00704.

### 3.4 miR-204-5p inhibitor alleviates the inhibitory effects on cell proliferation, cell cycle, migration, and invasion induced by LINC00704 depletion in PTC cells

**Figure 3:**
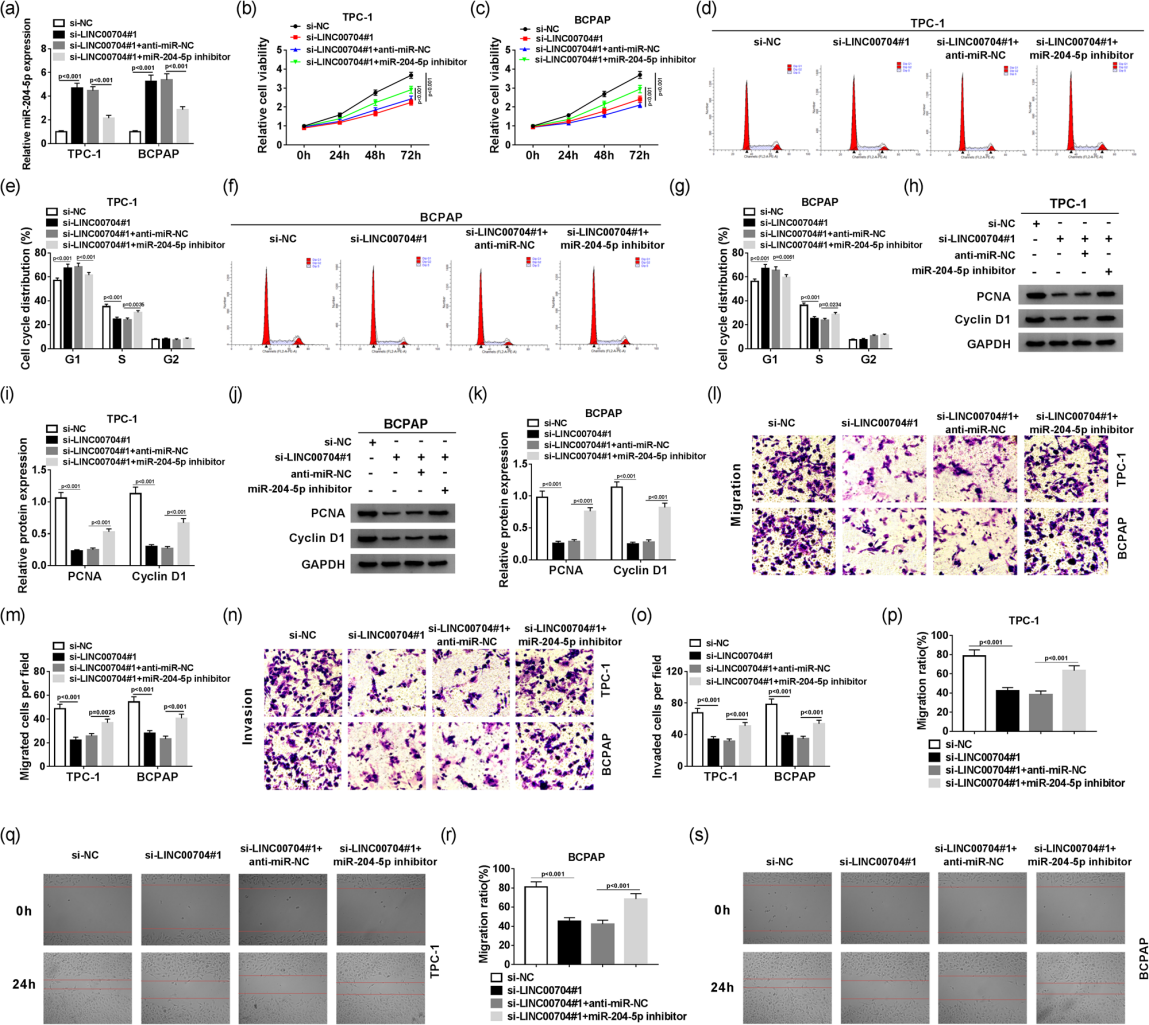
miR-204-5p is a target of LINC00704. (a) The level of miR-204-5p was measured by qRT-PCR in TPC-1 and BCPAP cells transfected with si-NC, si-LINC00704, si-LINC00704 + anti-miR-NC, or si-LINC00704 + miR-204-5p inhibitor. Cell viability (b and c), cell cycle (d-g), cell migration (l and m) and cell invasion (n and o) of transfected TPC-1 and BCPAP cells were assessed by MTT, flow cytometry, and transwell cell migration and invasion assays, respectively. (h–k) Protein levels of PCNA and cyclin D1 in transfected TPC-1 and BCPAP cells were assessed by Western blot assay. (p–s) Migration ratio was measured by wound-healing assay in transfected TPC-1 and BCPAP cells.

To further investigate the interaction between LINC00704 and miR-204-5p, qRT-PCR was conducted to measure the level of miR-204-5p in PTC cells co-transfected with si-LINC00704 and miR-204-5p inhibitor. The results showed that miR-204-5p was distinctly upregulated in si-LINC00704-transfected TPC-1 and BCPAP cells, while a miR-204-5p inhibitor attenuated this upregulation (Figure 3a). Furthermore, the MTT assay showed that cell viability was strikingly reduced in TPC-1 and BCPAP cells transfected with si-LINC00704, but the miR-204-5p inhibitor reversed the trend (Figure 3b and c). Meanwhile, flow cytometry results suggested that more cells were in the G1 phase due to the knockdown of LINC00704, which was abrogated by transfection of an miR-204-5p inhibitor in TPC-1 and BCPAP cells (Figure 3d–g). Moreover, LINC00704 silencing repressed the protein levels of proliferating cell nuclear antigen (PCNA; proliferation marker) and cyclin D1 (cell cycle marker), while the downregulation of miR-204-5p mitigated the effects in TPC-1 and BCPAP cells (Figure 3h–k), supporting the effects of LINC00704 and miR-204-5p on cell proliferation and cell cycle. Apart from that, the transwell assay showed that the migrated cells and invading cells were all strikingly reduced in TPC-1 and BCPAP cells transfected with si-LINC00704 but the miR-204-5p inhibitor reversed the trend (Figure 3l–o). Simultaneously, the wound-healing assay also proved that miR-204-5p knockdown could abolish the suppressive action of si-LINC00704 on the migration ratio in TPC-1 and BCPAP cells (Figure 3p–s). These data suggest that an miR-204-5p inhibitor could mitigate the inhibitory effects on cell proliferation, cell cycle, migration, and invasion in PTC cells.

### 3.5 HMGB1 negatively interacts with miR-204-5p

**Figure 4:**
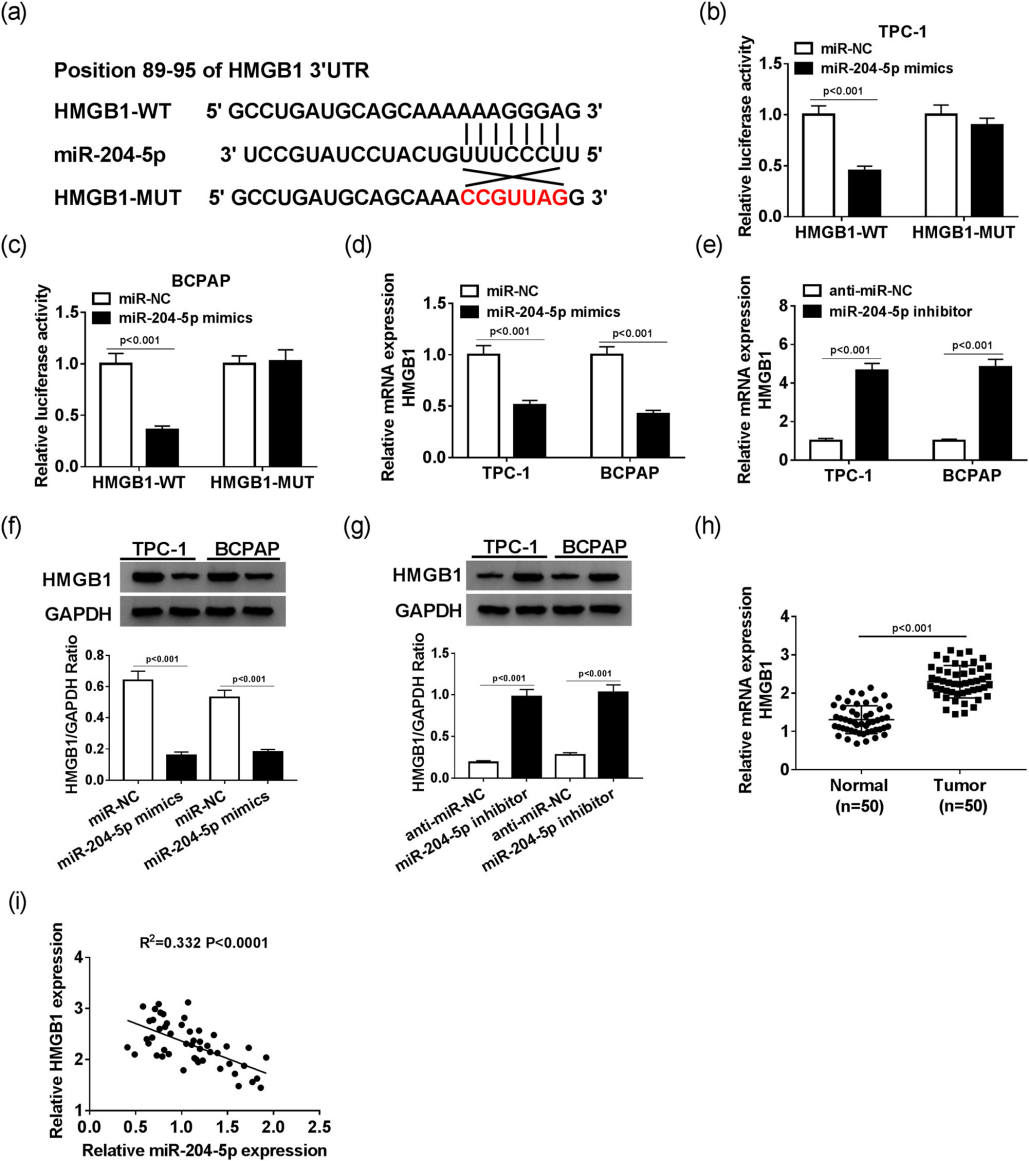
HMGB1 negatively interacts with miR-204-5p. (a) The putative complementary sequences between miR-204-5p and HMGB1 3′UTR. The luciferase activity of HMGB1-WT or HMGB1-MUT reporter in TPC-1 (b) and BCPAP (c) cells transfected with miR-204-5p mimics or miR-NC was assessed by dual luciferase reporter assay. The level of HMGB1 was detected by qRT-PCR in TPC-1 and BCPAP cells transfected with miR-204-5p mimics (d) or miR-204-5p inhibitor (e). The protein level of miR-204-5p was measured by Western blot in TPC-1 and BCPAP cells transfected with miR-204-5p mimics (f) or miR-204-5p inhibitor (g). (h) The level of HMGB1 in PTC tissues and adjacent normal tissues. (i) The correlation between HMGB1 and miR-204-5p.

To illustrate the biological mechanism of miR-204-5p, the putative target of miR-204-5p was identified with the TargetScan website. The search results suggested that miR-204-5p has complementary binding sites with the HMGB1 3′UTR (Figure 4a). The dual luciferase reporter assay indicated that miR-204-5p mimics significantly decreased the luciferase activity of HMGB1-3′UTR-WT related to that in miR-NC, while the luciferase activity of HMGB1-3′UTR-MUT was not obviously impaired in TPC-1 and BCPAP cells (Figure 4b and c). Furthermore, the mRNA and protein levels of HMGB1 both significantly decreased in TPC-1 and in BCPAP cells transfected with miR-204-5p mimics compared with that in miR-NC (Figure 4d and f); while the mRNA and protein levels of HMGB1 showed the opposite trend in TPC-1 and BCPAP cells transfected with an miR-204-5p inhibitor (Figure 4e and g). In addition, HMGB1 was apparently upregulated in PTC tissues in comparison with that in adjacent normal tissues (Figure 4h). The scatter diagram indicated that the level of HMGB1 was negatively linearly correlated with the level of miR-204-5p (Figure 4i). Taken together, HMGB1 negatively interacts with miR-204-5p.

### 3.6 HMGB1 overexpression reverses inhibitory effects on cell proliferation, cell cycle, migration, and invasion induced by miR-204-5p overexpression in PTC cells

**Figure 5:**
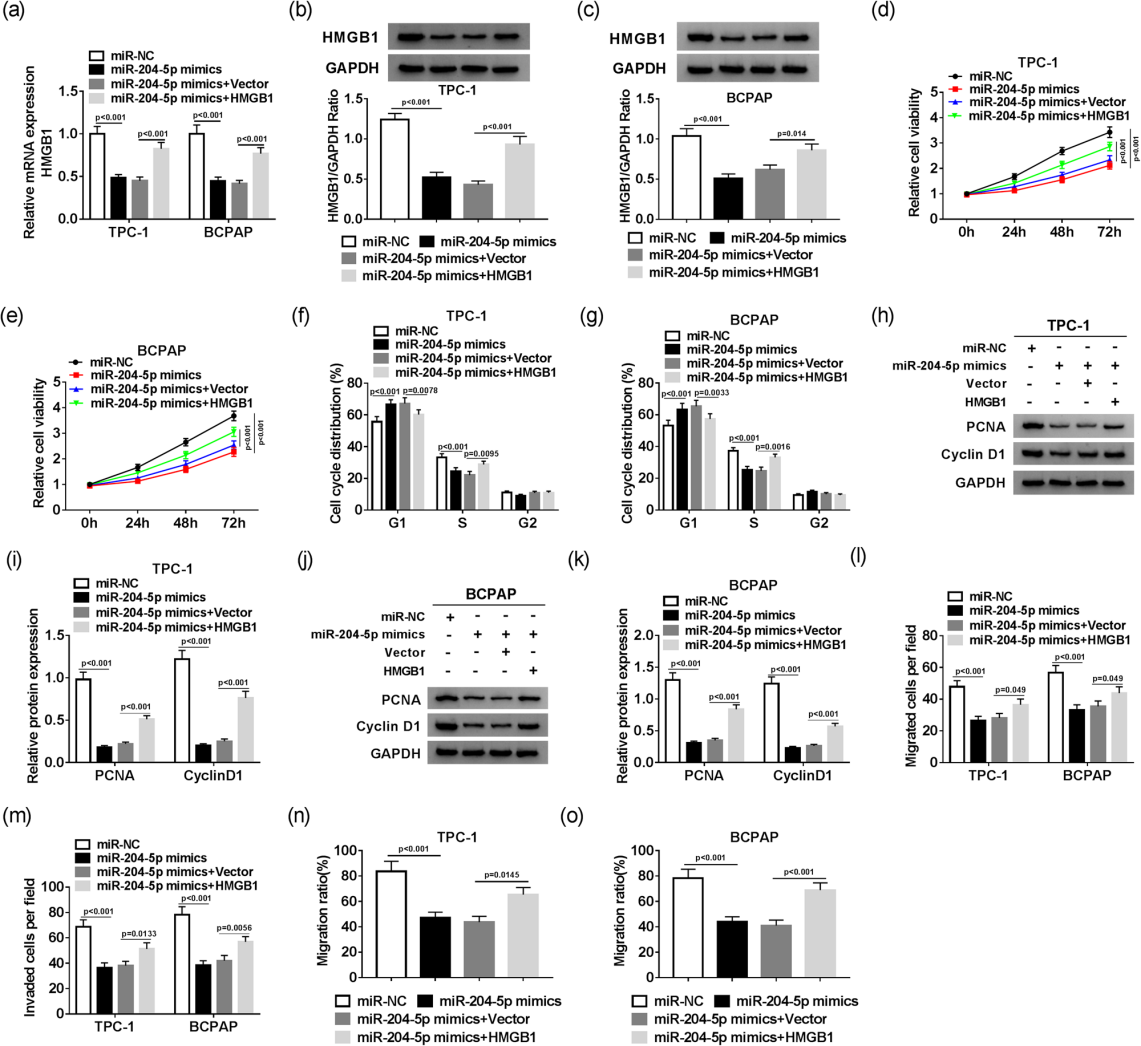
HMGB1 overexpression reverses the inhibitory effects on cell proliferation, cell cycle, cell migration and invasion induced by miR-204-5p overexpression in PTC cells. The relative mRNA (a) and protein (b and c) levels of HMGB1, cell viability (d and e), cell cycle (f and g), cell migration (l) and cell invasion (m), and migration ratio (n and o) in TPC-1 and BCPAP cells transfected with miR-NC, miR-204-5p mimics, miR-204-5p mimics + pcDNA or miR-204-5p mimics + pcDNA-HMGB1 were measured by qRT-PCR, Western blot, MTT, flow cytometry, transwell cell migration and invasion, and wound-healing assays, respectively. (h–k) Protein levels of PCNA and cyclin D1 in transfected TPC-1 and BCPAP cells were detected by Western blot assay.

To further research the interaction between miR-204-5p and HMGB1, miR-204-5p mimics and pcDNA-HMGB1 were co-transfected into TPC-1 and BCPAP cells. The qRT-PCR and Western blot assay results revealed that the mRNA and protein levels of HMGB1 were distinctly downregulated in TPC-1 and BCPAP cells transfected with miR-204-5p mimics, while the mRNA and protein levels of HMGB1 were oppositely affected in TPC-1 and BCPAP cells co-transfected with miR-204-5p mimics and pcDNA-HMGB1 in contrast to that in the corresponding matched controls (Figure 5a–c). Moreover, the MTT assay and flow cytometry assay results indicated that the overexpression of miR-204-5p inhibited cell viability and cell cycle in TPC-1 and BCPAP cells, whereas HMGB1 upregulation overturned these effects (Figure 5d–g). Furthermore, the changes in PCNA and cyclin D1 protein levels further demonstrated the regulatory effect of miR-204-5p and HMGB1 on cell proliferation and cell cycle (Figure 5h–k). Besides, the transwell assay indicated that migrated cells and invaded cells were greatly decreased in TPC-1 and BCPAP cells transfected with miR-204-5p mimics, while these inhibitory effects were mitigated by HMGB1 overexpression (Figure 5l and m). Synchronously, the migration ratio also showed a similar trend in TPC-1 and BCPAP cells (Figure 5n and o). These results demonstrated that HMGB1 overexpression weakened the inhibitory effects on cell proliferation, cell cycle, migration, and invasion induced by miR-204-5p overexpression in PTC cells.

### 3.7 LINC00704 silencing modulates HMGB1 low expression by sponging miR-204-5p in PTC cells

**Figure 6:**
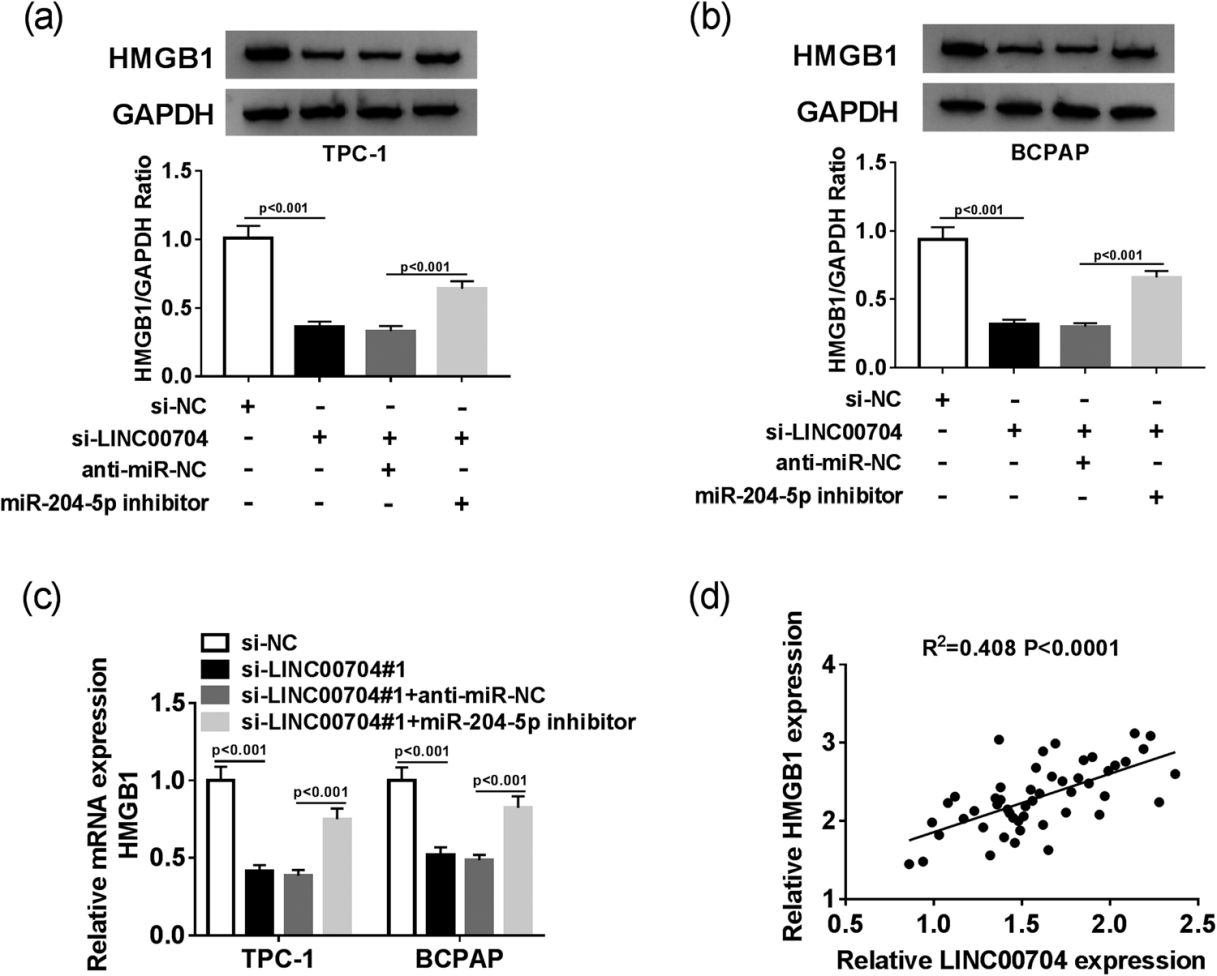
LINC00704 silencing modulates HMGB1 low expression by sponging miR-204-5p in PTC cells. The protein (a and b) and mRNA (c) levels of HMGB1 in TPC-1 and BCPAP cells transfected with si-NC, si-LINC00704, si-LINC00704 + anti-miR-NC, or si-LINC00704 + miR-204-5p inhibitor were detected by Western blot assay and qRT-PCR, respectively. (d) The correlation between HMGB1 and LINC00704. **P < 0.01.

Based on the above results, we further explored the interaction among LINC00704, miR-204-5p, and HMGB1. The qRT-PCR and Western blot assays indicated that the mRNA and protein levels of HMGB1 were both notably downregulated in si-LINC00704-transfected TPC-1 and BCPAP cells, but an miR-204-5p inhibitor mitigated these inhibitory effects on the mRNA and protein levels of HMGB1 (Figure 6a–c). In addition, the scatter diagram shows that the level of HMGB1 was positively linearly correlated with the level of LINC00704 (Figure 6d). These data suggest that LINC00704 knockdown downregulates HMGB1 by targeting miR-204-5p in PTC cells.

## 4 Discussion

PTC is a common endocrine system tumor. LncRNAs have been documented to participate in several processes in tumor progression. In this study, we aimed to explore the biological mechanism of action of LINC00704 in PTC. Based on the above results, we found that LINC00704 promotes cell proliferation, migration, and invasion in PTC via the miR-204-5p/HMGB1 axis.

LINC00704 has been reported to dysregulate expression and associated tumor progression in many cancers including PTC. Tracy et al. reported that the level of LINC00704 (mitotically associated long non-coding RNA) was obviously elevated in breast cancer tissues and cells (MDA-MB-231); the depletion of LINC00704 dramatically reduced cell proliferation and viability [18]. In this study, we verified that LINC00704 was highly expressed in PTC tissues and cells. LINC00704 levels correlated with the pathological characteristics of patients including tumor size, TNM stage, and lymph node metastasis. Moreover, LINC00704 knockdown inhibited cell proliferation, cell cycle, migration, invasion, and migration ratio in PTC cells. The above results are consistent with the previous study [6].

Recent studies demonstrated that miR-204-5p associates with cancer progression in many cancers. A report in breast cancer indicated that miR-204-5p was significantly downregulated in breast cancer tissues, and its overexpression repressed cell viability, proliferation, and migration capacity [19]. In fact, Liu et al. reported that the level of miR-204-5p was strikingly downregulated in PTC tissues and cell lines, and miR-204-5p overexpression restrained cell proliferation and induced cell cycle arrest and apoptosis in PTC cells [20]. In the present study, we validated that the level of miR-204-5p was strikingly decreased in PTC tissues and cells. LncRNAs have been reported as competing endogenous RNAs that can affect the levels of miRNAs and contribute to abnormal target mRNA expression. In the present study, we validated that the level of miR-204-5p was strikingly decreased in PTC tissues and cells. A dual luciferase reporter assay indicated that miR-204-5p directly interacts with LINC00704. MiR-204-5p was remarkably upregulated in TPC-1 and BCPAP cells, while the transfection of miR-204-5p mitigated these effects. In addition, an miR-204-5p inhibitor alleviated the inhibitory effects on cell proliferation, cell cycle, migration, and invasion caused by si-LINC00704.

HMGB1 has been identified as an essential contributor towards the initiation and progression of many kinds of cancers. For example, HMGB1 was found to be obviously upregulated in gastric cancer cells; HMGB1 silencing inhibited cell proliferation, colony formation, cell migration, and invasion and promoted cell apoptosis *in vitro* [21].

A study of PTC revealed that HMGB1 knockdown inhibited cell proliferation and metastasis in PTC cells *in vitro* and restrained xenograft tumor growth *in vivo* [16]. In this study, HMGB1 was markedly upregulated in PTC tissues. HMGB1 3′UTR was predicted to have complementary sequences with miR-204-5p. Then the dual luciferase reporter assay validated that HMGB1 is a direct target of miR-204-5p. The level of HMGB1 was downregulated in PTC cells transfected with miR-204-5p and upregulated in PTC cells transfected with an miR-204-5p inhibitor. Moreover, HMGB1 overexpression relieved the inhibitory effects on cell proliferation, cell cycle, migration and invasion in PTC cells induced by the miR-204-5p mimics. In addition, LINC00704 knockdown suppressed HMGB1 expression by sponging miR-204-5p.

## 5 Conclusion

In conclusion, our results indicate that LINC00704 and HMGB1 were upregulated and miR-204-5p was downregulated in PTC tissues and cells. LINC00704 modulates HMGB1 to promote cell proliferation, cell cycle, migration, and invasion in PTC by targeting miR-204-5p. The LINC00704/miR-204-5p/HMGB1 new regulatory pathway may provide a novel biomarker for PTC.
